# Antibody conjugates bispecific for intercellular adhesion molecule 1 and allergen prevent migration of allergens through respiratory epithelial cell layers

**DOI:** 10.1016/j.jaci.2015.01.006

**Published:** 2015-08

**Authors:** Christoph Madritsch, Julia Eckl-Dorna, Katharina Blatt, Isabella Ellinger, Michael Kundi, Verena Niederberger, Peter Valent, Rudolf Valenta, Sabine Flicker

**Affiliations:** a Division of Immunopathology, Department of Pathophysiology and Allergy Research, Center for Pathophysiology, Infectiology and Immunology, Medical University of Vienna, Vienna, Austria; b Department of Otorhinolaryngology, Medical University of Vienna, Vienna, Austria; c Division of Hematology and Hemostaseology, Department of Internal Medicine I, Medical University of Vienna, Vienna, Austria; d Division of Cellular and Molecular Pathophysiology, Department of Pathophysiology and Allergy Research, Center for Pathophysiology, Infectiology and Immunology, Medical University of Vienna, Vienna, Austria; e Institute of Environmental Health, Center of Public Health, Medical University of Vienna, Vienna, Austria

To the Editor:

It is becoming increasingly evident that intact epithelial barrier function is important for preventing allergens to reach tissues involved in allergic inflammation.^1^ Therefore it is tempting to speculate that strategies that either strengthen epithelial barrier function or prevent allergen from crossing the epithelium might have therapeutic potential.

In this study we produced an antibody conjugate bispecific for intercellular adhesion molecule 1 (ICAM1) and a major respiratory allergen (ie, the major grass pollen allergen Phl p 2)^2^ termed P2/ICAM1 to investigate whether such a conjugate can inhibit the migration of allergens through respiratory cell layers and reduce the activation of the inflammatory cells underneath.

ICAM1 was used as a target molecule to anchor allergen-specific antibodies on human bronchial epithelial cells because it is expressed on the surfaces of respiratory epithelial cells, especially under inflammatory conditions, and has a low turnover rate.^3^ Furthermore, ICAM1 is a major target molecule for cellular entry of human rhinovirus (HRV) strains, which are implicated in asthma exacerbations,^4^ and has been reported to be highly expressed on the respiratory epithelium of allergic patients.^5^

Antibody conjugates were formed through streptavidin-biotin coupling (ie, conjugation) by using different ratios of the individual components (P2/ICAM1: 1:1, 1:0.5, and 1:0.25; see the Methods section in this article's Online Repository at www.jacionline.org). P2/ICAM1, but not a conjugate formed with a Phl p 5–specific antibody, reacted specifically with recombinant Phl p 2 and ICAM1, as shown by means of ELISA (see the Methods section and Fig E1, Fig E2 in this article's Online Repository at www.jacionline.org). Furthermore, P2/ICAM1 bound specifically to the respiratory epithelial cell line 16HBE14o- and immobilized Phl p 2 on the cells, as shown by using fluorescence-activated cell sorting (FACS) and immunofluorescence microscopy, respectively (Fig 1 and see the Methods section and Fig E3 in this article's Online Repository at www.jacionline.org). Images in Fig 1 show colocalization (Fig 1, *A*, yellow, merge) of Phl p 2 (red, Alexa Fluor 568) and P2/ICAM1 (green, Alexa Fluor 488) on the cell surface. When either Phl p 2 (Fig 1, *B*) or Phl p 2–specific rabbit antibodies (Fig 1, *C*) were omitted, no binding of Alexa Fluor 568 goat anti-rabbit IgG was observed. When P2/ICAM1 was added to the cells, it was detected with Alexa Fluor 488 goat anti-mouse IgG specific for αICAM1 mouse IgG (Fig 1, *A-C*, green). When P2/ICAM1 was omitted, detection antibodies did not bind (Fig 1, *D*). No cell staining was found when only secondary antibodies were applied (data not shown). P2/ICAM1 remained bound to 16HBE14o- cell surfaces for up to 72 hours at 37°C (see Fig E4 in this article's Online Repository at www.jacionline.org).

**Fig 1:**
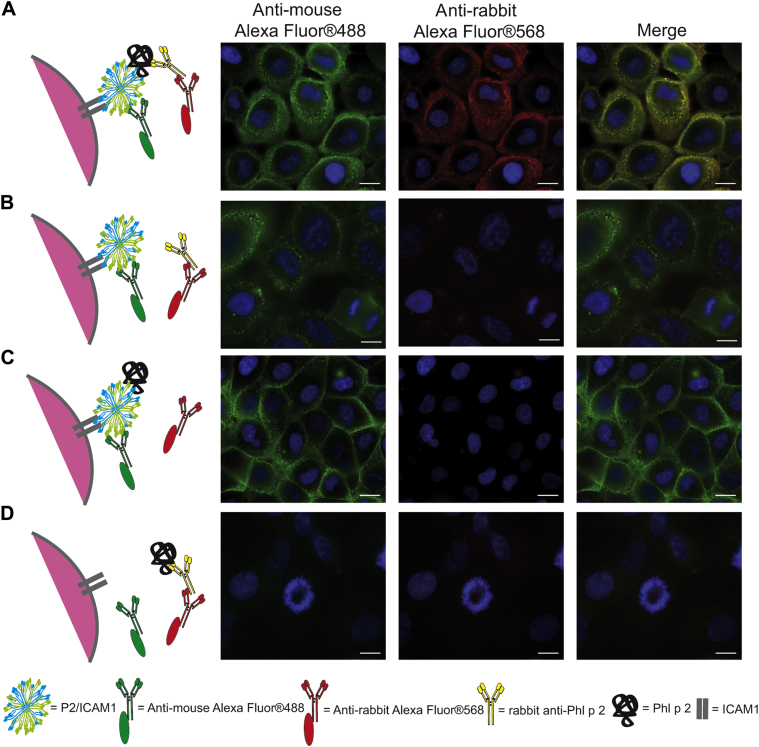
Visualization of P2/ICAM1 and Phl p 2 on 16HBE14o- cells by means of immunofluorescence microscopy. Images (**A**-**D**) show cells incubated with different combinations of reactants (*left margin* and *bottom*), which were stained with Alexa Fluor 488–labeled anti-mouse antibodies *(green, left column*) and Alexa Fluor 568–labeled anti-rabbit antibodies *(red, middle column)* to visualize αICAM1-mouse IgG and Phl p 2, respectively. Nuclei were stained with 4′, 6-Diamidino-2-Phenylinodole, Dihydrochloride *(blue)*, and merged images are shown in the *right column*. *White bars* = 20 μm.

In a subsequent series of experiments, we demonstrated that P2/ICAM1 can prevent the apical-to-basolateral penetration of Phl p 2 but not of the birch pollen allergen Bet v 1, a similarly sized but not related allergen, through a layer of the cultured respiratory epithelial 16HBE14o- cells by using a well-established Transwell culture system (Costar Corning Incorporated, Corning, NY; see Fig E5, Fig E6 in this article's Online Repository at www.jacionline.org), as described in the Methods section in this article's Online Repository.^6^ In the experimental Transwell model allergen concentrations were tested that, according to studies analyzing the release of grass pollen allergens during pollen seasons, might naturally occur on the mucosa.^7^ Furthermore, we showed that P2/ICAM1 also reacted specifically with natural grass pollen–derived Phl p 2 (data not shown).

In the Transwell experiments we found that the apical addition of P2/ICAM1 prevented the penetration of Phl p 2 over the full period of analysis (ie, for 72 hours) into the basolateral compartment (see Fig E5, *A*, gray bars, +) when compared with conditions without addition of P2/ICAM1 (Fig E5 *A*, gray bars, −). When Phl p 2 and P2/ICAM1 conjugates were omitted, no Phl p 2 signal was detected (see Fig E5, *A*, no Phl p 2, 72 hours). Additionally, no relevant penetration of P2/ICAM1–Phl p 2 complexes into the basolateral wells was found (Fig E5, *B*, gray bars, +). The addition of the P2/ICAM1 conjugate alone did not affect the integrity of the epithelial layers when resistance was measured at 24, 48, and 72 hours (data not shown).

Finally, we tested whether a reduction of transepithelial allergen migration by P2/ICAM1 has an effect on basophil activation to assess the migration of Phl p 2, capable of activating mast cells or basophils. Results from basophil activation tests (see the Methods section and Fig E7 in this article's Online Repository at www.jacionline.org) performed with samples from 3 independent Transwell experiments with basophils from 3 representative allergic patients are shown in Fig 2. The extent of basophil activation induced with basolateral samples showed a significant and consistent reduction for samples taken from cultures in which P2/ICAM1 had been added to the apical compartments (Fig 2, gray bars, +) compared with those in which P2/ICAM1 conjugates had been omitted (Fig 2, gray bars, −). This effect was observed at each of the analyzed time points. Basophil activation induced by samples taken from the apical compartments (Fig 2, black bars) was comparable and almost identical with that obtained with basolateral samples from cell-free preparations (Fig 2, no cells). Basophil activation was not observed when Phl p 2 was absent from the cultures (Fig 2, no Phl p 2). Concentrations of soluble ICAM1 in 16HBE14o- cell cultures were around 1 ng/mL in apical wells and not detectable in basolateral wells in the experiments and had no influence on allergen-induced basophil activation (data not shown).

**Fig 2:**
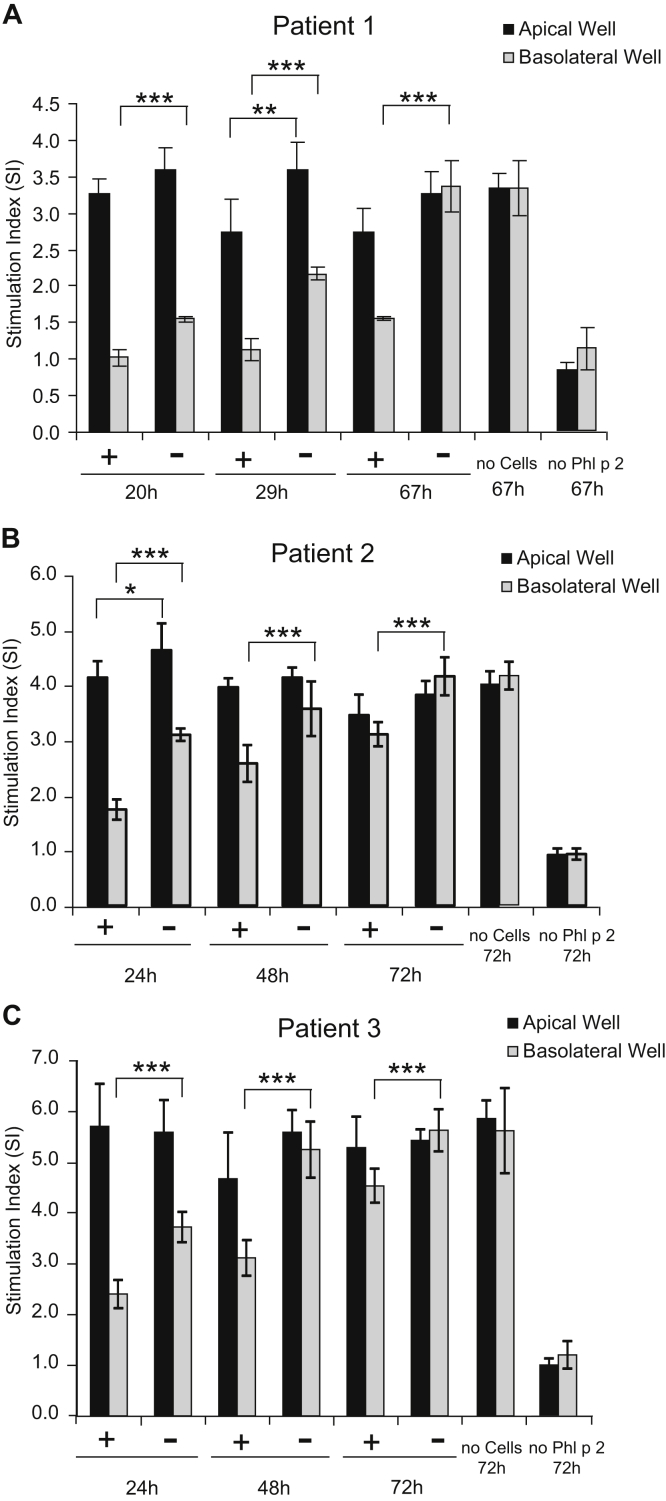
Inhibition of transepithelial migration of Phl p 2 by P2/ICAM1 conjugates leads to decreased basophil activation with basolateral samples. Samples obtained from apical and basolateral compartments of Transwell cultures were incubated with blood samples from 3 patients allergic to Phl p 2 **(A-C)**, and basophil activation was measured by determining upregulation of the surface marker CD203c on basophils by using flow cytometry. CD203c upregulation expressed as the stimulation index is displayed on the *y-axis*. Results are means of triplicates, and *error bars* indicate SDs. *P < .05, **P < .01, and ***P < .001, ANOVA and linear contrasts.

Our experiments thus demonstrate that it is possible to use antibody conjugates bispecific for ICAM1 and a major respiratory allergen to inhibit allergen migration through respiratory cell layers and to reduce the activation of inflammatory cells underneath.

Because increasing numbers of human high-affinity antibodies specific for a large variety of clinically relevant allergens are becoming available,^8^ it should be possible to engineer recombinant bispecific antibody constructs for a variety of respiratory allergens that can be combined to protect patients against several different sources for which the major allergens have been identified. The topical application of the conjugates to target organs of allergic inflammation might help prevent allergens from intruding into the tissues and, subsequently, from inducing allergic inflammation and boosting allergen-specific IgE responses. For example, the conjugates can be administered to the nose, eyes, buccal mucosa, and eventually lung to treat respiratory, conjunctival, and oral allergic symptoms.

Taking the finding into consideration that anti–ICAM1 antibodies were shown to inhibit rhinovirus infections and HRV-induced inflammation, one might also speculate that antibody conjugates that are bispecific for ICAM1 and allergens might have a synergistic protective effect in allergic patients with HRV-induced asthma exacerbations.^9^

## Figures and Tables

**Fig E1:**
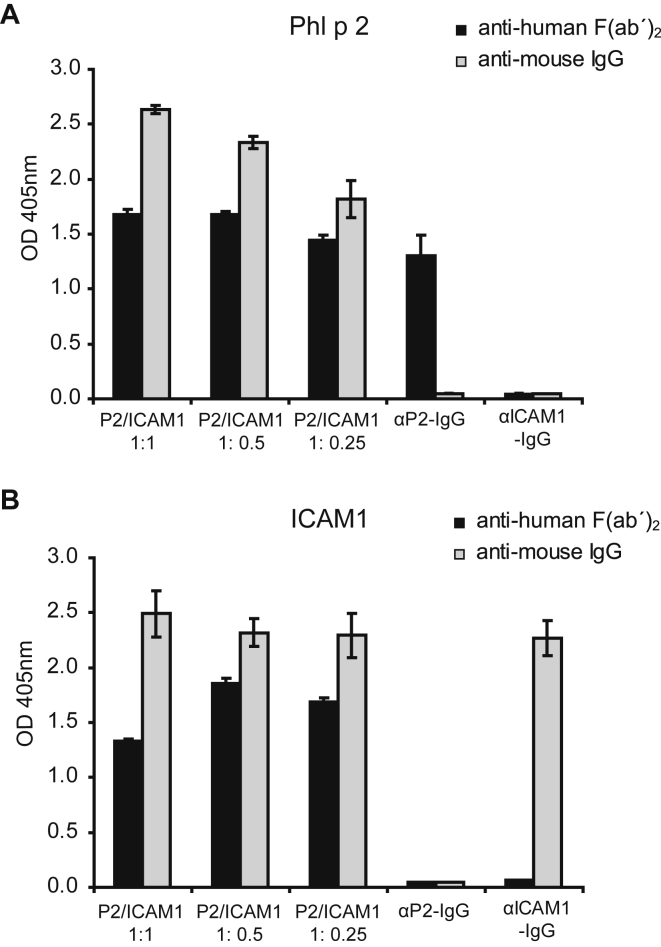
Reactivity of P2/ICAM1 with Phl p 2 and ICAM1 in ELISAs. Conjugates formed with different ratios of anti–Phl p 2 and anti-ICAM1 (*x-axes*: P2/ICAM1, 1:1, 1:0.5, and 1:0.25), anti–Phl p 2 *(αP2-IgG)*, or anti–ICAM1 *(αICAM1-IgG)* alone were tested for reactivity with Phl p 2 **(A)** or ICAM1 **(B)** and detected with anti-human F(ab′)_2_ antibodies *(black bars)* or anti-mouse IgG *(gray bars)*. OD values *(y-axes)* corresponding to bound P2/ICAM1 conjugates are shown as means of triplicates ± SDs.

**Fig E2:**
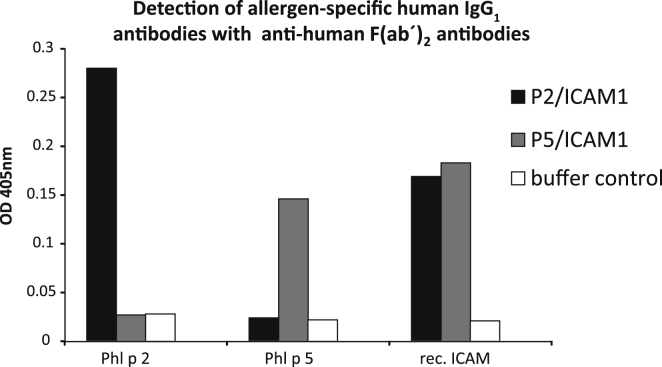
Binding of the bispecific antibody conjugates P2/ICAM1 and P5/ICAM1 to the immobilized allergens Phl p 2 and Phl p 5 and recombinant ICAM1 in ELISAs. Bound P2/ICAM1 and P5/ICAM1 were detected with alkaline phosphatase–conjugated anti-human F(ab′)_2_ antibodies. Buffer containing 0.5% BSA was applied as a negative control (buffer control). One representative experiment of 3 is shown.

**Fig E3:**
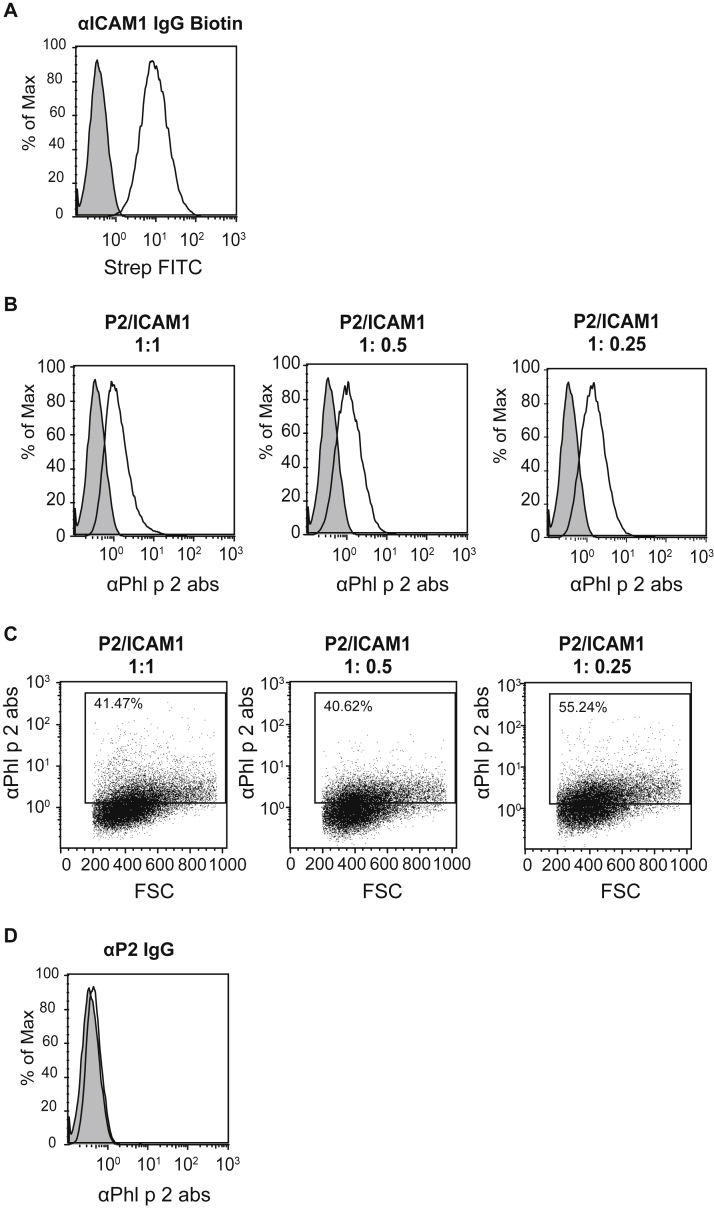
Detection of ICAM1 or P2/ICAM1–captured Phl p 2 on respiratory epithelial cells by using flow cytometry. **A,** ICAM1 was detected on the surfaces of 16HBE14o- cells by using ICAM1–specific antibodies *(white graph)* or an isotype control *(gray graph)*. *FITC*, Fluorescein isothiocyanate. **B,** Cells preincubated with different ratios of P2/ICAM1 (1:1; 1:0.5; and 1:0.25) and Phl p 2 were probed for cell-bound Phl p 2 with specific rabbit antibodies *(white graphs)* or the isotype control *(gray graphs)*. **C,** Scatter plots showing percentages of Phl p 2–reactive cells with 3 different ratios of P2/ICAM1. **D,** Cells incubated with Phl p 2–specific human IgG_1_ antibodies, followed by Phl p 2 and Phl p 2–specific rabbit antibodies. One representative experiment of 3 independent experiments is shown.

**Fig E4:**
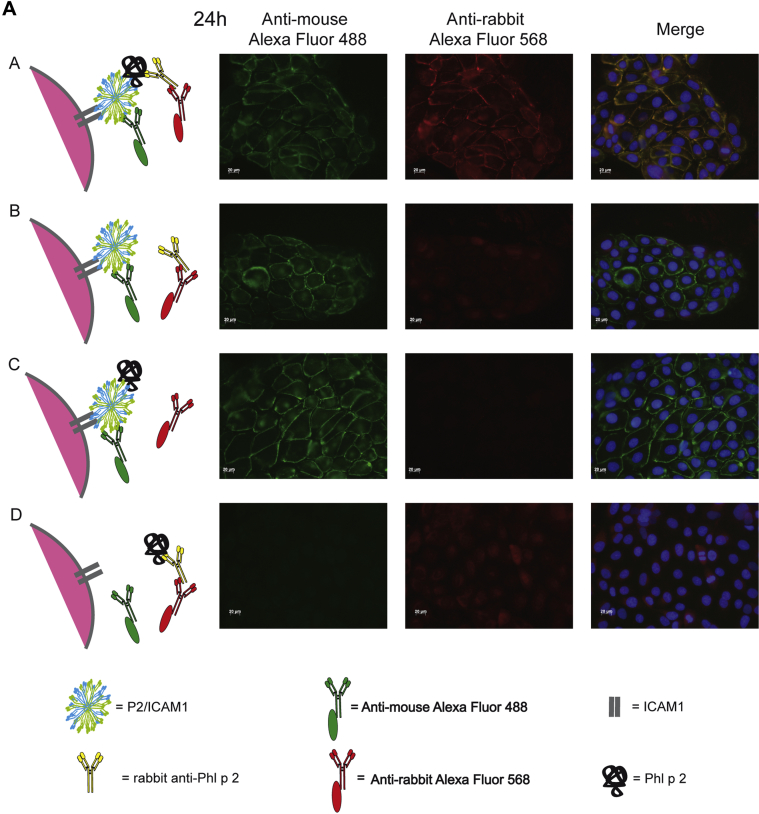

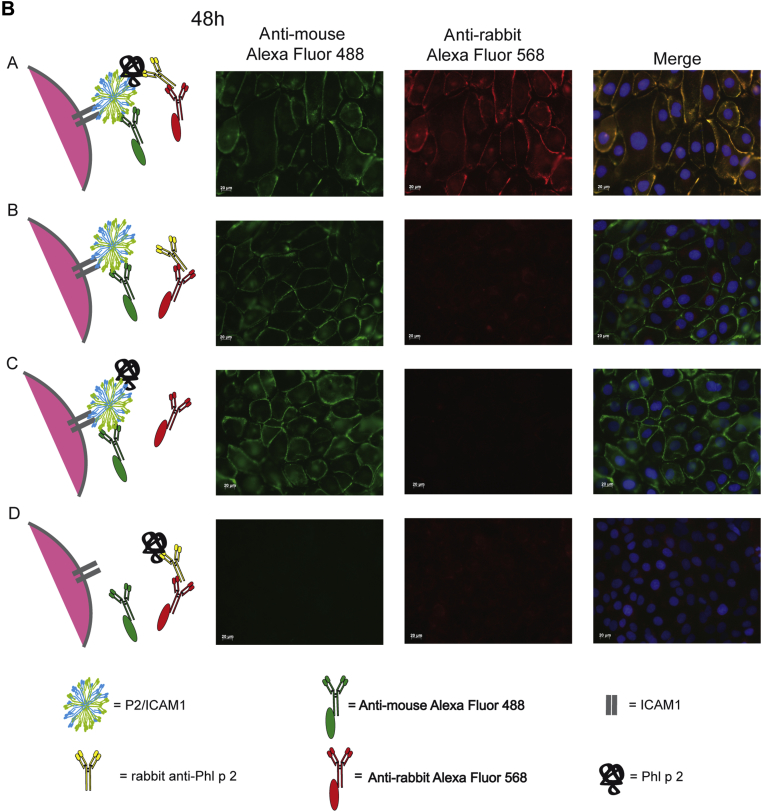

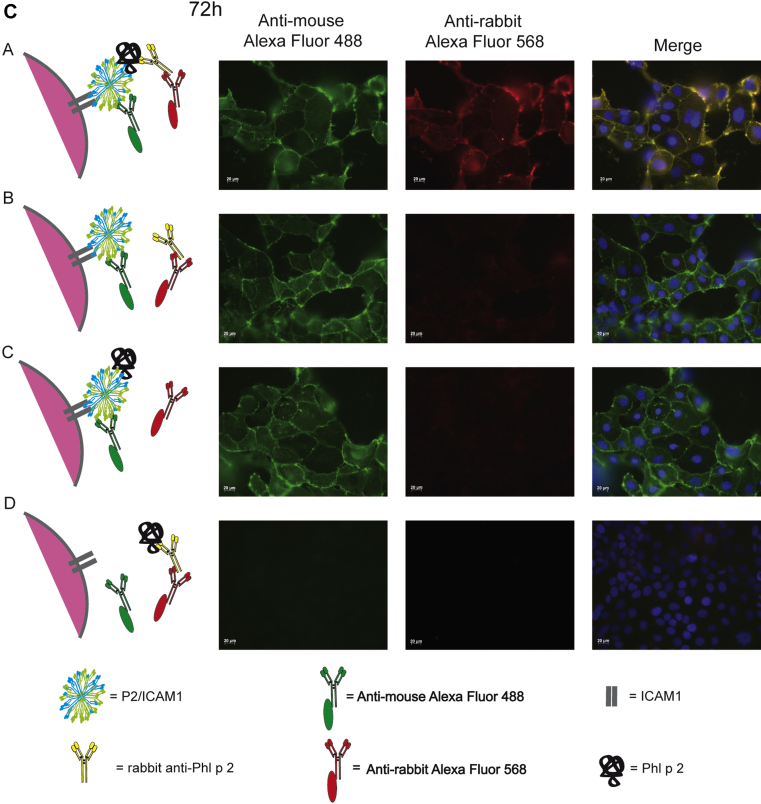
Visualization of P2/ICAM1 and Phl p 2 on 16HBE14o- cells by using immunofluorescence microscopy over a time course of 24 **(A)**, 48 **(B)**, and 72 **(C)** hours. Images showing cells incubated with different combinations of reactants (*left margin* and *bottom*), which were stained with Alexa Fluor 488–labeled anti-mouse antibodies *(green, left column)* and Alexa Fluor 568–labeled anti-rabbit antibodies (*red*, *middle column*) to visualize αICAM1–mouse IgG and Phl p 2, respectively. Nuclei were stained with 4′, 6-Diamidino-2-Phenylinodole, Dihydrochloride *(blue)*, and merged images are shown in the right column. *White bars* = 20 μm.

**Fig E5:**
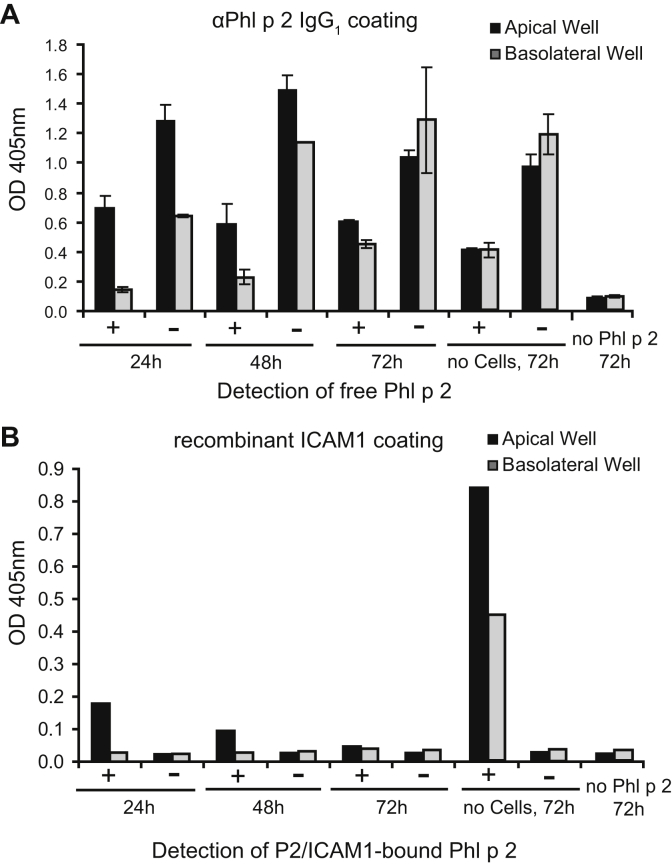
Cell-bound P2/ICAM1 conjugates inhibit transepithelial migration of Phl p 2. **A,** ELISA detection of free Phl p 2 in apical *(black bars)* and basolateral *(gray bars)* compartments of cultured 16HBE14o- cell monolayers that had been preincubated with *(+)* or without *(−)* P2/ICAM1 conjugates. Concentrations of free Phl p 2 corresponding to OD values *(y-axis)* were measured at different time points (*x-axis*: 24, 48, and 72 hours) in wells with or without cells *(no cells)*. In the negative control Phl p 2 was omitted *(no Phl p 2)*. **B,** Simultaneous detection of P2/ICAM1–Phl p 2 complexes in the same samples as in Fig E5, *A*. Shown is one of 3 independent experiments producing comparable results.

**Fig E6:**
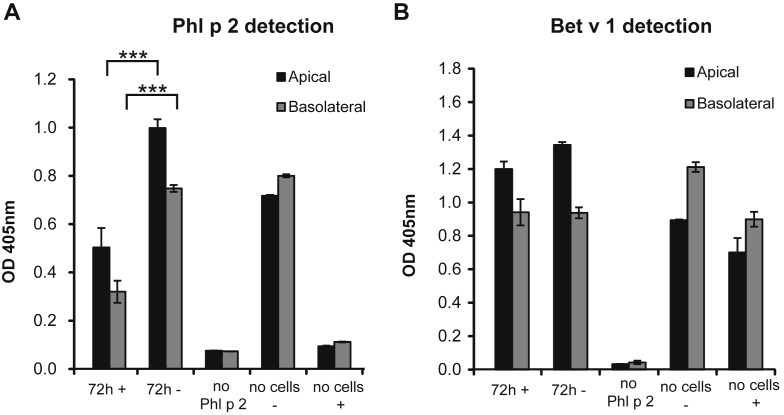
Cell-bound P2/ICAM1 conjugates inhibit transepithelial migration of Phl p 2 **(A)** but not of the unrelated allergen Bet v 1 **(B)**. ELISA detection of free Phl p 2 (Fig E6, *A*) or free Bet v 1 (Fig E6, *B*) in apical *(black bars)* and basolateral *(gray bars)* compartments of cultured 16HBE14o- cell monolayers that had been preincubated with *(+)* or without *(−)* P2/ICAM1 conjugates. Concentrations of free Phl p 2 or Bet v 1 corresponding to OD values *(y-axis)* were measured at 72 hours in wells with or without cells *(no cells)*. In the negative control Phl p 2 was omitted *(no Phl p 2)*. ***P < .001, significant difference.

**Fig E7:**
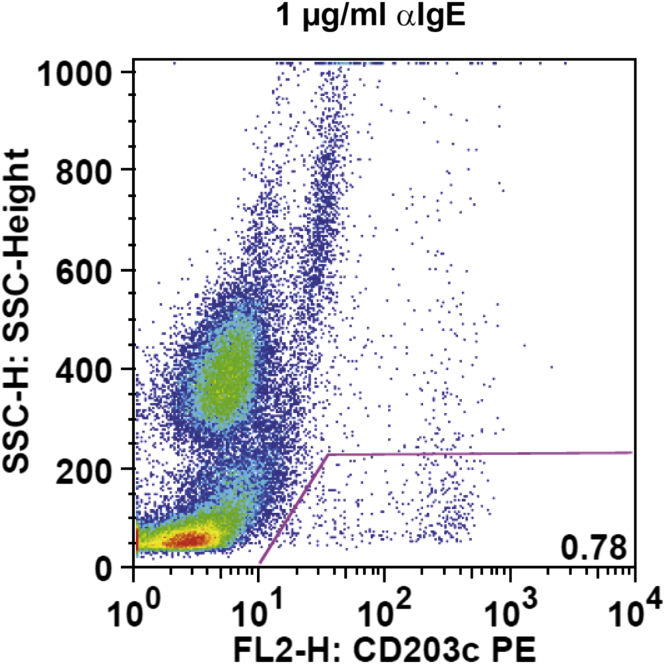
Basophils were detected on the basis of side-scatter characteristics and expression of CD203c and analyzed with Paint-a-gate in the FlowJo program. Basophils are defined as CD203c^+^ cells in all samples. *PE*, Phycoerythrin; *SSC*, side scatter.

## Methods

### Antibodies and antibody conjugation

A Phl p 2–specific IgE Fab fragment was previously isolated from an allergic patient, as described by Steinberger et al.^E1^ This Fab was converted into a fully human IgG_1_ antibody^E2^ and stably expressed in CHO-K1 cells (Madritsch and Flicker, unpublished data). This Phl p 2–specific IgG_1_ was purified by means of Protein G affinity chromatography (Thermo Fisher Scientific, Pierce, Rockford, Ill). The Phl p 2–specific IgG_1_ antibody was conjugated with Lightning-Link Streptavidin (Innova Biosciences, Cambridge, United Kingdom). A Biotin-labeled ICAM1–specific mouse IgG_1_ antibody was purchased from Abcam (Cambridge, United Kingdom). Three different ratios (1:1, 1:0.5, and 1:0.25) of the Streptavidin-labeled Phl p 2–specific human IgG_1_ and the Biotin-labeled ICAM1–specific mouse IgG_1_ were coincubated for at least 1 hour at room temperature to form a bispecific antibody conjugate, which was termed P2/ICAM1.

### ELISA evaluation of the binding specificities of P2/ICAM1 conjugates

ELISA plates (Nunc Maxi-Sorp, Roskilde, Denmark) were coated with recombinant ICAM1 (R&D Systems, Minneapolis, Minn), recombinant Phl p 2 (5 μg/mL), and grass pollen extract containing 5 μg/mL natural Phl p 2 or recombinant Phl p 5 as an unrelated control allergen (5 μg/mL; Biomay AG, Vienna, Austria) in 100 mmol/L NaHCO_3_ (pH 9.6). Plates were incubated for 1 hour at 37°C, washed twice with PBS containing 0.05% (vol/vol) Tween-20 (PBST), and saturated with PBST containing 3% (wt/vol) BSA. P2/ICAM1 (1 μg/mL) was applied overnight at 4°C. Bound P2/ICAM1 was detected either with horseradish peroxidase–conjugated rat anti-mouse IgG_1_ antibodies (BD PharMingen, San Diego, Calif) or alkaline phosphatase–conjugated goat anti-human F(ab′)_2_ antibodies (Pierce), both diluted 1:5000 in PBST containing 1% (wt/vol) BSA. OD measurements were carried out on ELISA reader Spectramax Plus (Molecular Devices, Sunnyvale, Calif) at 405 nm. Results are means of triplicates. Error bars indicate SDs.

### Flow cytometry

Aliquots of approximately 150,000 16HBE14o- cells were incubated with 1 μg of P2/ICAM1 in 50 μL FACS buffer (PBS supplemented with 2% wt/vol BSA) for 30 minutes on ice. After washing, aliquots of 1 μg Phl p 2 in 50 μL FACS buffer were added, and cells were again incubated for 30 minutes on ice. After further washing, cells were incubated with Phl p 2–specific rabbit antibodies and an Alexa Fluor 488–labeled goat anti-rabbit antibody (Molecular Probes, Life Technologies, Eugene, Ore). Biotin-conjugated anti–ICAM1 antibody was visualized with streptavidin conjugated to Alexa Fluor 488 (Molecular Probes). 7-AAD dye (Beckman Coulter, Brea, Calif) was used for staining of dead cells. Controls included the biotin-conjugated anti–ICAM1 antibody isotype control (mouse IgG_1_ MOPC-21, Abcam) and the Phl p 2–specific antibody conjugated to streptavidin, followed by Phl p 2 and Phl p 2–specific rabbit antibodies. Cells were analyzed on a FC500 Flow Cytometer (Beckman Coulter) counting at least 30,000 cells per sample in duplicates and were evaluated with FlowJo software (version 7.2.5; TreeStar, Ashland, Ore).

### Immunofluorescence microscopy

16HBE14o- cells, which had been cultured as described,^E3^ were seeded on ibiTreat tissue culture–treated μ-dishes (Ibidi GmbH, Munich, Germany) and grown for 2 days to approximately 50% confluence. Cells were washed twice with PBS, cooled to 4°C, and further processed at 4°C unless stated otherwise. Antibody-allergen complexes were formed by means of coincubation of 5 μg of P2/ICAM1 and 1 μg Phl p 2 in 300 μL PBS for 30 minutes at room temperature and subsequently incubated with the cells at 4°C for 30 minutes. For the time course (24-72 hours) immunofluorescence experiments shown in Fig E4, antibody-allergen complexes were incubated with cells at 37°C for 24, 48, and 72 hours. Unbound complexes were removed by washing with PBS, and cells were then incubated with Phl p 2–specific rabbit antibodies diluted 1:1000 in PBS. Cells were washed with PBS and fixed with 4% formaldehyde solution for 20 minutes at room temperature, and the remaining aldehyde groups were quenched with 50 mmol/L ammonium chloride in PBS for 10 minutes. Unspecific binding sites were blocked with 10% goat serum in PBS overnight at 4°C. Alexa Fluor 488 goat anti-mouse IgG (Molecular Probes) diluted 1:1000 in PBS containing 10% goat serum was added to the cells for 1 hour at room temperature to detect cell-bound P2/ICAM1. Phl p 2 bound to P2/ICAM1 was visualized with Alexa Fluor 568–labeled goat anti-rabbit IgG (Molecular Probes) applied at a dilution of 1:1000 in PBS for 1 hour at room temperature. Finally, nuclei were stained with 1 μg/mL 4′, 6-Diamidino-2-Phenylinodole, Dihydrochloride (Molecular Probes) in PBS. Cells were washed twice with PBS between individual incubations. Staining with fluorescent antibodies alone was done to assess background signals. Controls omitting either Phl p 2, Phl p 2–specific rabbit antibodies, or P2/ICAM1 were included. Wide-field fluorescence imaging of fixed cells was carried out with a Zeiss Axio Observer Z1 inverted fluorescence microscope equipped with an oil immersion 40 × lens (Pan Apochromat, 1.4 NA; Carl Zeiss, Oberkochen, Germany) and the Zeiss AxioVision Software package (release 4.8).

### ELISA differentiating free Phl p 2 from P2/ICAM1–bound Phl p 2 in culture samples

The Phl p 2–specific human IgG_1_ was coated (1 μg/mL in 100 mmol/L NaHCO_3_ [pH 9.6]) on ELISA plates, which were washed and blocked, as described above, to measure free Phl p 2 in culture samples. Phl p 2 was then detected with rabbit anti–Phl p 2 antibodies (1:1000).^E4^ Bound rabbit antibodies were visualized with a horseradish peroxidase–conjugated donkey anti-rabbit antibody diluted 1:2000 (GE Healthcare, Little Chalfont, United Kingdom).

Recombinant ICAM1 (1 μg/mL in 100 mmol/L NaHCO_3_ [pH 9.6]) was coated on ELISA plates, which were washed and blocked, as described above, to detect P2/ICAM1–bound Phl p 2. Cell-culture supernatants were applied, and P2/ICAM1–bound Phl p 2 was detected with rabbit anti–Phl p 2 antibodies, as described above. Results represent means of duplicates, with error bars indicating SDs.

The human sICAM1 Platinum ELISA (extra sensitive) from eBioscience (Vienna, Austria) was used to measure levels of soluble ICAM1 in culture samples.

### Cell culture and Transwell experiments

The epithelial cell line 16HBE14o- derived from human bronchial surface epithelial cellsE5, E6 was cultivated, as previously described.^E3^ Baseline transepithelial electrical resistance ranged consistently from 110 to 120 Ω/cm² for Transwells filled with medium without cells and was measured with an ohm voltmeter (Millipore, Bedford, Mass). When cells reached a transepithelial electrical resistance value of 400 Ω/cm² (baseline subtracted), IFN-γ (50 ng/mL; PeproTech, Rocky Hill, NJ) was added in all cultures to allow detectable allergen migration. Aliquots of 5 μg of P2/ICAM1 (ratio 1:0.25) were applied to the apical chambers for 3 hours at 37°C. Supernatants were removed, and 10 ng of Phl p 2 or 10 ng of Bet v 1 in 0.5 mL of medium was added to the apical compartment for 20 to 72 hours at 37°C. According to the calculations of Schäppi et al,^E7^ 10 ng of allergen per well was supposed to be an amount in the physiologic range. The effect of P2/ICAM1 on Phl p 2 apical-to-basolateral transepithelial migration was assessed by comparing P2/ICAM1-1–treated wells with untreated control wells. In addition, the transmigration of Phl p 2, as well as that of P2/ICAM1–bound Phl p 2, through Transwell membranes without cells was assessed. Samples from the apical and basolateral compartments were tested for the presence of free Phl p 2, P2/ICAM1–bound Phl p 2, or both by means of ELISA.

### Basophil activation assays

Basophil activation was assessed *in vitro* by measuring upregulation of CD203c expression. Heparinized blood from patients with grass pollen allergy with IgE antibodies against Phl p 2 were obtained after informed consent was given. Blood aliquots (90 μL) were incubated with either samples from the Transwell experiments or for control purposes with an anti-IgE mAb (1 μg/mL; Immunotech, Vaudreuil-Dorion, Quebec, Canada) or PBS for 15 minutes at 37°C. Allergen-induced upregulation of CD203c was calculated from mean fluorescence intensities (MFIs) obtained with stimulated (MFIstim) and unstimulated (MFIcontrol) cells and expressed as the stimulation index (MFIstim/MFIcontrol).^E8^ Cells were analyzed by means of 2-color flow cytometry on a FACScan (BD Biosciences, San Jose, Calif). The gating strategy is shown in Fig E7.
